# Polysaccharide Hydrogel-Assisted Biosensing Platforms for Point-of-Care Use

**DOI:** 10.3390/bios15010013

**Published:** 2025-01-02

**Authors:** Sang-Uk Kim, Young Jun Kim, Tae Hee Lee

**Affiliations:** 1 Korea Science and Technology Holdings, Ltd., 593, Daedoek-Daero, Yuseong-Gu, Daejeon 34112, Republic of Korea; 2 School of Integrative Engineering, Chung-Ang University, 4, Heukseok-Ro, Dongjak-Gu, Seoul 06974, Republic of Korea; 3 Department of Biomedical Laboratory Science, Daegu Health College, Chang-ui Building, 15 Yeongsong-ro, Buk-gu, Daegu 41453, Republic of Korea

**Keywords:** polysaccharide, hydrogel, point-of-care testing, biosensor, paper-based analytical device, microfluidics, stimuli-responsive behavior, alginate, agarose, chitosan

## Abstract

Point-of-care (POC) use is one of the essential goals of biosensing platforms. Because the increasing demand for testing cannot be met by a centralized laboratory-based strategy, rapid and frequent testing at the right time and place will be key to increasing health and safety. To date, however, there are still difficulties in developing a simple and affordable, as well as sensitive and effective, platform that enables POC use. In terms of materials, hydrogels, a unique family of water-absorbing biocompatible polymers, have emerged as promising components for the development of biosensors. Combinations of hydrogels have various additional applications, such as in hydrophilic coatings, nanoscale filtration, stimuli-responsive materials, signal enhancement, and biodegradation. In this review, we highlight the recent efforts to develop hydrogel-assisted biosensing platforms for POC use, especially focusing on polysaccharide hydrogels like agarose, alginate, chitosan, and so on. We first discuss the pros and cons of polysaccharide hydrogels in practical applications and then introduce case studies that test different formats, such as paper-based analytical devices (PADs), microfluidic devices, and independent platforms. We believe the analysis in the present review provides essential information for the development of biosensing platforms for POC use in resource-limited settings.

## 1. Introduction

Point-of-care (POC) testing is a laboratory test performed where care is needed most [1,2,3]. The representative analytes and subjects of interest in POC testing include glucose, cholesterol, coagulation, cardiac markers, hematology, blood gas, pregnancy, and infertility [4,5]. These analytes and subjects share the common need for frequent, repetitive, and continuous monitoring. Some of these are closely related to circumstances in which rapid determination is required for personal or public safety. Recently, we witnessed the importance of POC testing once again during the coronavirus disease 2019 (COVID-19) pandemic [6]. The supply of centralized laboratories with specialized equipment (e.g., polymerized chain reaction tests) and highly trained personnel did not match the explosively increased testing demands. In the meantime, rapid antigen kits that could be either self-operated or administered with the help of others at home or in the workplace helped to relieve the burden of urgent testing demands [7]. In addition, rapid antigen kits were employed as a decentralized testing strategy, providing rapid check-ups at hospitals, airport terminals, train stations, and so on. During the pandemic, numerous biosensing studies for detecting severe acute respiratory syndrome coronavirus 2 (SARS-CoV-2) focused specifically on developing a platform for POC use in resource-limited settings [8]. This was also because early diagnosis on the basis of a decentralized testing strategy was the one and only way to constrain these fast-spreading and highly infectious viruses. POC testing is often considered to be a specific theme of biomedical sensing, but its basic concept is also necessary in many other applications. For example, there is also a need for on-site testing in environmental monitoring, where quick check-ups on water, air, and soil quality are required at multiple sites at regular time intervals. These substantial testing numbers cannot be satisfied by the capacity of centralized laboratories. Even if this was possible, this approach would be inefficient in terms of time and cost. Similar to the use of rapid antigen kits, on-site detection of targets of interest in water, air, or soil could provide early awareness and warn of potential risks, enabling early action to reduce environmental impacts. Similarly, food safety is another important field that requires POC use. There are several requirements for developing POC tests [8]. Firstly, of course, sensitivity and selectivity are of the utmost importance and need to be sufficient to exclusively recognize the target of interest. Secondly, as sample-in and answer-out systems, rapid output of the results from the sample input is a key factor in POC testing. Third, affordability, as well as robustness and stability, is another key factor for end-users. Fourth, equipment-free and user-friendly tests are also essential to enable mass-scale testing.

In light of the aforementioned considerations, researchers have increasingly been paying attention to hydrogels—hydrophilic polymeric networks with extraordinary water-absorbing properties—for the development of an effective platform for POC testing, both on their own or as part of the platform. This special class of polymer has an extraordinary ability to absorb water compared to its insoluble polymeric components, offering soft and biocompatible materials [9]. In addition, the dynamic change in the volume of the hydrogel as a result of surrounding conditions makes them a source of smart materials [10]. Therefore, hydrogels have enough potential to develop efficient biosensing platforms in various ways.

In this review, we provide a comprehensive analysis of the recent developments in POC testing with the help of polysaccharide hydrogels. These naturally occurring polymeric materials are abundant and biocompatible. However, their benefits also come with inherent limitations, such as large variations in sources, inconsistent functions, and low stability. It should be noted that the properties of natural hydrogels struggle to compete with those of highly engineered synthetic hydrogels, which usually show superior performance based on versatile functionality in most applications. Nevertheless, this subject is interesting, as efforts have been made to overcome their shortcomings in engineering applications in order to take advantage of these cost-effective and environmentally friendly materials. We believe these hydrogel-assisted approaches share the long-term objectives of developing and using POC biosensors in resource-limited settings.

## 2. Polysaccharide Hydrogels

Polysaccharides are long-chain carbohydrates that link monosaccharides with glycosidic linkages [11,12]. They are the largest biopolymer family on Earth and form structural components and energy storage units in plants and microorganisms [13]. The representative polysaccharides include cellulose, chitin/chitosan, starch, alginate, gum, pectin, carrageenan, and so on. These biopolymers exist everywhere around us and thus can easily be obtained at affordable cost. In addition, these naturally occurring biopolymers are safe, biocompatible, and degradable materials. Historically, mankind has exploited polysaccharide hydrogels in diverse fields, including food production, processing, and related areas. We can recognize them around us as thickening agents, gelling agents, emulsifiers, stabilizers, or texturizers in various food products [14]. Furthermore, polysaccharides can be ideal food packaging film and edible film due to barrier properties and non-toxicity, keeping the contained food safe for a long time [15].

There have been extensive studies to develop hydrogel-assisted biomedical assays. Liquid biopsy, which obtains the disease-related biomarkers from the biofluid, is one representative example [16,17,18]. As an isolation support or an encapsulation matrix for biomarkers, hydrogels can be utilized to isolate and recover biomarkers [19]. For example, alginate-based hydrogel was exploited as degradable isolation support for both circulating tumor cells and exosomes [20]. Considering the importance of marker recovery in liquid biopsy research, mild and effortless recovery based on the degradation ability of polysaccharide hydrogels can be a rational approach. In the meantime, hydrogel can be an ideal matrix to protect valuable markers from harsh conditions. Encapsulation is often defined as the action of enclosing certain substances into another material as a physical barrier (i.e., capsule) [21]. The main purpose of this measure is to protect the encapsulated substance from the environment. Some of the advantages of these studies are worth sharing and are still effective in the development of POC tests.

## 3. Hydrogel-Assisted Biosensing Platforms

Hydrogels are beneficial when integrating biosensing platforms for the following advantages. (a) They are excellent adsorbents because of their high porosity and high surface area. (b) Their networks act like filters during the absorption or desorption of water. The large-sized background molecules cannot penetrate the inner networks. In contrast, the component inside hydrogels is preserved from the external fluid without disassociation. (c) Their hydrophilic polymeric networks are biocompatible supports for biorecognition elements (e.g., antibodies, aptamers, or other affinity molecules). (d) The regularly confined space between networks can be worked as a sort of well-ordered reactor. (e) Hydrogels have stimuli-responsive properties that actively react to the surrounding environment or the amount of reaction. For example, the dynamic changes in volume can be an indicator. The distance-based readout is an extraordinary feature of a hydrogel-assisted platform [22]. Because the volume change in hydrogel is possible in both microscale and macroscale, the differences can be quite distinguishable according to the dimension. This means that we can convert the analyte-induced changes in volume into the function of analyte concentration. It is one of the feasible concepts of instrument-free detection methods. However, its control is often restricted by the environmental conditions. Also, the sensitive nature of the hydrogel against temperature and moisture can reduce the accuracy. Similarly, the mechanical properties of the hydrogel can be used to monitor the degree of reaction. In the case that antibodies are immobilized on the polymeric networks and the analytes can pass through the inner networks, the antigen–antibody reaction induces deformation of the hydrogels, accelerating the further reaction [23].

Another example is the signal enhancement using the swelling–shrinking ability of the hydrogels. When the sensing components for signal generation (e.g., gold nanoparticle, fluorophore, or chromogenic reagents) are included in the hydrogel matrix, the swelling state of hydrogels is an interesting strategy to enhance the output signal per unit volume, including color, fluorescence, or even plasmonic enhancement. In surface-enhanced Raman scattering (SERS) spectroscopy-based studies, the shrinkage of the hydrogel containing plasmonic nanomaterials enables the generation of strong and controllable signals by the increment of hotspot density [24,25]. However, the difficulties in the consistent arrangement of plasmonic nanomaterials inside the hydrogel matrix can cause signal uniformity and reproducibility issues. Also, the effect of environmental conditions, including temperature and humidity, at measurement can reduce the controllability of a hydrogel-based platform.

However, naturally occurring polymers have both advantages and disadvantages in biosensing applications. First of all, they have large variations in sources and inconsistent functions, which make them difficult to use. Second, low mechanical, chemical, and thermal stability is a key obstacle to their engineering and applications. They are not as rigid as synthetic hydrogels under load-bearing positions, and their sensitivity to temperature and moisture can be a drawback that affects their structural integrity in sensors. Third, the formation as well as degradation of these hydrogels are mostly uncontrollable. This issue causes inconsistent objects and functions. The aging or leaching effects of polymeric components also reduce the shelf-life of the resulting platforms. Fourth, particularly in electrical or electrochemical biosensors, these polymeric macromolecules are likely to act like insulators due to low conductivity and low adhesion, reducing the output signals unless there is a hybridization of conducting materials. Therefore, the proper hybridization with other synthetic polymers or incorporation with nanomaterials (e.g., graphene oxide) has been investigated [26,27].

**Figure 1:**
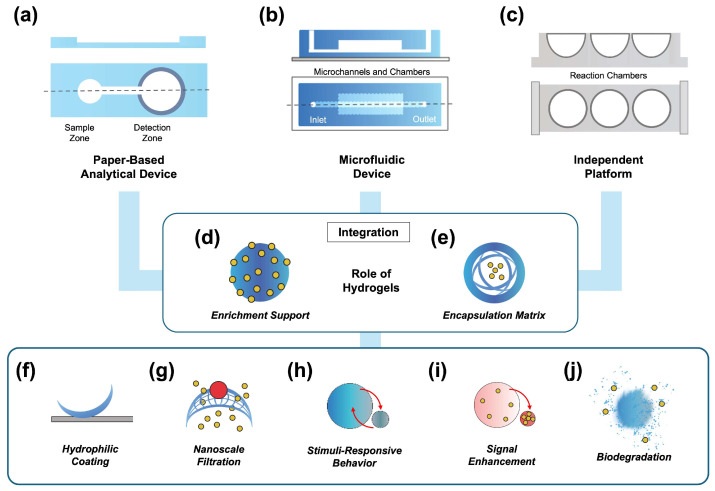
Hydrogel-assisted biosensing platforms for point-of-care use. (**a**–**c**) Representative format of POC biosensors: (**a**) paper-based analytical devices (PAD), (**b**) microfluidic device, (**c**) independent platform; (**d**,**e**) Role of hydrogels in the system; (**f**–**j**) Various functions of the hydrogels in the system.

In the following sub-sections, we summarize recent studies on developing biosensing platforms for POC use with the assistance of polysaccharide hydrogels. We will discuss the existing formats for POC testing, such as paper, microfluidics, and circuit boards, and then discuss the use of hydrogel as an independent format. Figure 1 describes the representative POC format and the role of the hydrogels on the device/platform.

### 3.1. Integration with Paper-Based Analytical Devices (PADs)

Paper-based analytical devices (PADs) are representative formats in POC testing because of their simple operation and rapid response time. Paper is the simplest substrate that possesses lightness, flexibility, and low thickness [28]. In the economic aspect, PADs cost less in production, owing to inexpensive raw materials and mass-producible processes. In addition, they require fewer samples and also consume fewer reagents compared to traditional assays [29]. On the other hand, PADs have several limitations in terms of practical applications. First, their sensitivity and specificity are often unsatisfactory due to methodological limitations, including diffusion-dependent sample development and naked-eye confirmation. Although there have been several concepts to enhance performance with the help of instruments (e.g., SERS), the benefits of the instrument-free method are somewhat degraded under these combinational approaches. Second, paper-based systems have a possibility of contamination and are limited in the simultaneous detection of multiple analytes.

Although PADs have a long history in the development of user-friendly testing platforms, the incorporation of hydrogel into PADs has just recently come into the spotlight. In fact, hydrogels share numerous advantages with papers, which are made of cellulose fibers. Hydrogels are basically 3-dimensional polymeric networks that can adsorb the liquid samples. Like the running of the samples on the PADs, the diffusion of the samples into the polymeric networks of the hydrogel can happen. The difference here is the capacity to contain liquid. As their nomenclature implies, hydrogels are hydrophilic semi-solid and can adsorb extraordinary amounts of water compared to their dried weight. For these reasons, hydrogels on the PADs can play a role in absorbing samples or collecting signals.

Table 1 describes recent studies of the integration of polysaccharide hydrogels with PADs for the development of biosensing platforms for POC use. The evaluation criteria in the table are closely related to the major requirements of POC use, such as sensitive detection, rapid response, and stability. Xu et al. reported a simple one-step colorimetric PAD for the detection of glucose in blood samples [30]. Glucose is the most significant example of analytes in POC use requiring frequent monitoring via convenient and repetitive tests. The device consisted of a wax-modified hydrophobic zone and an alginate hydrogel-coated hydrophilic zone. The latter contained enzymes (glucose oxidase, GOx) and chromogenic reagents (3,3′,5,5′-tetramethylbenzidine, TMB). Particularly, this concept is advantageous because whole blood samples are loaded without dilution. The porous structure of the hydrogel provides filter-like performance, repelling blood cells and biomacromolecules. The authors validate the feasibility of the method using whole blood samples obtained before and after meals.

**Figure 2:**
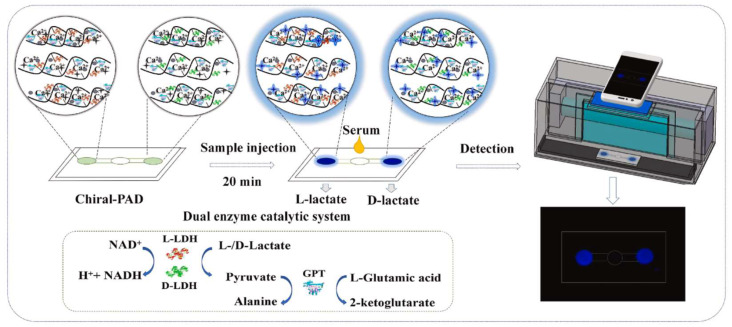
Schematic diagram for chiral assays using the hydrogel chiral-PAD and the detection and data acquisition using a smartphone (reprinted from [36], copyright (2023), with permission from Elsevier).

The gelation of the hydrogel-based platform itself can serve as an indicator. As shown in Figure 2, Wang et al. presented a paper-based hydrogel platform using the dual-signal determination of L-lactate [31]. The proposed platform is based on the diffusion diameter on the paper as a change in viscosity of the alginate solution along with the enzymatic transformation. The pre-gel alginate solution contains lactate dehydrogenase (LDH), Rhodamine B (RB), and calcium carbonate (CaCO_3_). The L-lactate, an organic acid closely related to digestive function, was converted to pyruvate and hydrogen ions using LDH, thus inducing gelation by the ionization of CaCO_3._ At the same time, pyruvate accelerated the degradation of RB, fading its pink color. The visual distance reading was conducted with a vernier caliper after a 10-min reaction, while the color change was read by RGB analysis using a smartphone. The limit of detection (LoD) of visual distance reading and smartphone-assisted colorimetric analysis was 0.03 μM and 0.07 μM, respectively. They mentioned that the importance of L-lactate quantification is to control the acidity of the yogurt or other fermented food products.

The introduction of the hydrogel not only enhances the stability of these biomolecules, but also increases the total amount of the biomolecules. It is a meaningful approach because nitrocellulose is not a favorable surface for biomolecules. Tang et al. reported the increment of immobilization capacity of biomolecules and proved that this approach holds great potential for enhancing test performance [32]. In the demonstration using HPV, they achieved a ten-fold enhancement of detection sensitivity. However, they also pointed out that the original porosity of the nitrocellulose membrane can be changed during the incorporation of chitosan.

In the meantime, microfluidic paper-based analytical devices (μPADS) are a category of PADs that take advantage of microfluidics. μPADs are different from common microfluidic systems because they do not require the instrument to drive sample flow. Like usual PADs, μPADs are just dependent on the capillary force inside the paper. Gabriel et al. proposed an enhancement method using chitosan for a microfluidic paper-based device [33]. The device, fabricated by a stamping process, consisted of a central sample loading zone and eight circularly arranged detection zones. Thereafter, the detection zones were modified using a chitosan solution. They proved that more effective reaction zones that adsorb more enzymes were fabricated by the chitosan modification. The LoDs for glucose and uric acid was 23 μM and 37 μM, respectively. Finally, they verified the analytical reliability and accuracy of the device with a comparison of spectrophotometry techniques.

**Table 1:** Representative studies of the integration of polysaccharide hydrogels with paper-based analytical devices (PADs) for the development of biosensing platforms for point-of-care use.

Polysaccharide Hydrogel	Target Analyte	Sensing Components	Confirmation	Matrix	Detection Range	Limit of Detection	Response Time	Stability Test	Reference
Agarose	Dengue Virus RNA	Au Nanoparticle (AuNP)	Naked Eye	Buffer	N/A	50 copies	N/A	N/A	2016 [34]
Alginate	Glucose	CdZnTeS QD /GOx	Smartphone	N/A	100 to 1000 μM	1.1 μM	10 m	N/A	2021 [35]
	Glucose	GOx /HRP/TMB	Smartphone	PBS	0.36 to 15 mM	0.12 mM	12 m	40 d	2021 [30]
				Whole Blood	2.20 to 15 mM	(0.12 mM) **			
	L-lactate D-lactate	LDH/ GPT	Smartphone	PBS	0.1 to 3.0 mM 0.01 to 0.5 mM	30.0 ± 0.7 μM 3.0 ± 0.2 μM	20 m	40 d	2023 [36]
	L-lactate	Rhodamine B /LDH/CaCO_3_	Visual Distance	N/A	0.1 to 15 mM	0.03 μM	10 m	N/A	2024 [31]
			Smartphone	N/A	0.3 to 15 mM	0.07 μM	N/A		
Chitosan	Glucose	GOx/HRP/ 4-AAP/DHBS	Naked Eye	PBS	0.1 to 5 mM	23 μM	15 m	10 d	2016 [33]
	Uric Acid	Uricase/HRP/ 4-AAP/DHBS	Naked Eye	PBS	0.1 to 5 mM	37 μM			
	Hepatitis B Virus DNA	AuNP	Naked Eye	N/A	2.5 to 20 nM	0.05 nM	15 m	N/A	2020 [32]

Abbreviation: Quantum Dot (QD), Glucose oxide (GOx), Horseradish peroxidase (HRP), 3,3′,5,5′-tetramethylbenzidine (TMB), Lactate dehydrogen-ase (LDH), Glutamic pyruvic transaminase (GPT), 4-aminoantipyrine (4-AAP), (3, 5-dichloro-2-hydroxybenzenesulfonate) (DHBS), Phosphate-buffered saline (PBS), Not applicable (N/A). ** This is an estimate assuming all other conditions are same to PBS environment.

### 3.2. Integration with Microfluidic Devices

Microfluidic devices are another important format for POC testing [37]. These small and portable systems can integrate several sequential sample processes from the sample pretreatment to results acquisition, including separation, extraction, dilution, mixing, washing, and chemical reaction, following the flow of the samples [38]. Because most of the entire process is conducted inside the microfluidic channels, it is relatively free from sample contamination or unexpected handling errors throughout the multiple procedures; thus, test repeating at identical conditions allows us to obtain consistent and reliable results. Although microfluidic devices require complicated fabrication procedures like photolithography, there is freedom of design on the basis of the demand.

Table 2 describes recent studies of the integration of polysaccharide hydrogels with microfluidics for the development of biosensing platforms for POC use. The first advantage of hydrogels here is the modification of the hydrophobic surface of the traditional microfluidic devices. Silicon elastomers like poly(dimethylsiloxane) (PDMS) have excellent formability, but their hydrophobic surface is prone to non-specific binding and is not suitable for biomolecule functionalization. In this context, hydrogels can work as a coating layer to perform a specific function or reduce non-specific binding. Hatch et al. demonstrate microfluidics with an engineered hydrogel layer [39]. The microposts having a diameter of 100 μm inside the microfluidic channel were modified by engineered alginate containing four-arm poly(ethylene glycol)-conjugated antibodies and alginate. The increase in antibodies enhances the isolation of endothelial progenitor cells from whole human blood while the hydrogel layer reduces the background signal. Considering the hydrophobic nature of PDMS, modifying it with a hydrophilic polymer is likely to be an effective way to control the inner surface of the device.

Hydrogels themselves can be utilized to construct microchannels with a simple procedure at a low cost. Although their capability to fabricate the structure with high resolution is unsatisfactory compared to the PDMS, hydrogel-based microfluidics do not require additional force to drive the flow of the samples (e.g., syringe pump). Furthermore, the extraordinary water permeability of hydrogel is advantageous in concentrating samples. Li et al. presented agarose-based tree-shaped microchannels to focus pathogens [40]. They fabricated the device using the standard soft lithography, and PDMS was only utilized for the impermeable barrier to contain bacterial suspension. Thus, the sample flow was driven by the capillary force and water permeability of the agarose gels. The samples containing bacteria cells were delivered to the reservoir with a recovery efficiency of over 90%. The seven orders of enrichment in cell density at the condition of 10^3^ cells make the device useful when handling large volumes of biofluids like urine and blood plasma.

**Figure 3:**
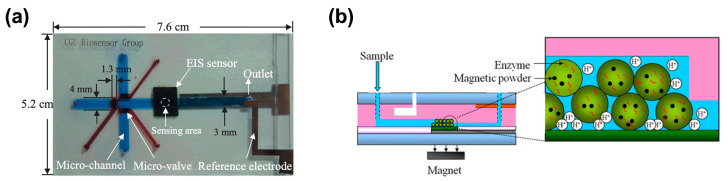
Integrating solid-state sensor and microfluidic devices using enzyme-carrying alginate microbeads. (**a**) Photograph of assembled microfluidic chip and (**b**) schematic diagram of enzyme-carrying alginate microbeads that are immobilized on an electrolyte–insulator–semiconductor sensor surface by means of step-like obstacle and external magnetic field (reprinted from [41], copyright (2013), with permission from Elsevier).

Hydrogels can be utilized as carriers for reaction reagents in the specific microfluidic chambers. Lin et al. demonstrated a microfluidic chip containing magnetic powder-containing alginate microbeads for the multiplexed detection of glucose, urea, and creatinine [41]. As shown in Figure 3, the microbeads were positioned on the surface of the electrolyte-insulator-semiconductor (EIS) sensor by magnetic force. When the samples were introduced to the reaction chamber, samples reacted with the enzyme-containing microbeads, causing the changes in voltage signal. The role of alginate beads here is to prompt the reaction through the effect as a step-like obstacle and to protect the activity of the enzyme. The detection range of the device is 2–8 mM, 1–16 mM, and 0.01–10 mM for glucose, urea, and creatinine, respectively. Lastly, they validated the performance of the present microfluidic chip with the commercial kits.

In the meantime, microfluidic devices have been considered useful in hydrogel synthesis, owing to their ability to control small and uniform volumes of fluid inside the channels [42]. In the case of the chemically cross-linkable hydrogels, we can fabricate micrometer-sized hydrogel beads by designing a structure that occurs that meets the drops of pre-gel solution and cross-linking solution in the oil-containing microchannels. In the case of photo cross-linkable hydrogels, this research can control the pre-gel solution in the channel, thus generating the cross-linked hydrogels via photo exposure on the channels. These micro-sized hydrogel beads are highly uniform and suitable for use in the development of efficient and reproducible tests.

**Table 2:** Representative studies of the integration of polysaccharide hydrogels with microfluidics for the development of biosensing platforms for point-of-care use.

Polysaccharide Hydrogel	Target Analyte	Sensing Components	Confirmation	Matrix	Detection Range	Limit of Detection	Response Time	Stability Test	Reference
Agarose	AST ALT ALP	Au-LDO TMB/H_2_O_2_	Smartphone	Buffer	15 to 150 U/L 10 to 180 U/L 5 to 70 U/L	15 U/L 10 U/L 5 U/L	30 m	12 h-tracking	2022 [43]
Alginate	Glucose	GOx	LCR meter	PBS	2 to 8 mM	0.0625 mM	20 s	N/A	2013 [41]
	Urea	Urease			1 to 16 mM	0.001 mM			
	Creatinine	Creatinine Deiminase			0.01 to 10 mM	0.001 mM			
	Lactate	QD, Lactate Oxidase	Smartphone	Tris Buffer	0.1 to 1.0 mM	1.25 μM	15 m	15 d	2021 [44]
	Glucose	GOx/HRP /TMB/TiO_2_	Digital Camera	Artificial Sweat	10 to 1000 μM	7.7 μM	16 m	N/A	2023 [45]

Abbreviations: Au-decorated CoAl-layered double oxide (Au/LDO), aspartate transaminase (AST), alanine transaminase (ALT), and alkaline phosphatase (ALP), 3,3′,5,5′-tetramethylbenzidine (TMB), Glucose oxide (GOx), Quantum Dot (QD), Horseradish peroxidase (HRP), Phosphate-buffered saline (PBS), Not applicable (N/A).

### 3.3. Independent Hydrogel Platforms

Hydrogels themselves can be operated as independent platforms without integrating any other existing format of analytical platforms. One of the strong features of the hydrogels is easy gel formation and shaping. It is possible to fabricate the hydrogels by controlling the gelation process. For example, hydrogels can have the form of sheets, disks, blocks, and spheres/beads without any supports to match the objective of applications [19]. The use of common reaction plates (e.g., 24-, 48-, or 96-well plates) allows us to utilize the advantages of well-established existing techniques. Unlike the PADs or microfluidic systems, this approach is highly advantageous in running multiple tests simultaneously. Therefore, the development of a hydrogel-only POC testing format is also a reasonable choice.

Table 3 describes the recent studies of polysaccharide hydrogel-based platforms for the development of biosensing platforms for POC use. Punjabi et al. developed hydrogel nanospheres to detect bacteria in the samples. The authors fabricated chitosan nanoparticles and conjugated lectins using ionic gelation for the rapid detection of the bacteria [46]. Crystal violet, a positively charged dye, is known to be bound to the cell walls of the bacteria. When the bacteria were introduced to the vial, agglutination was caused, and the outcome was recognizable by the naked eye. Lastly, they designed and produced a simple but very effective POC testing kit consisting of two vials with a Pasteur pipet in custom packaging.

**Figure 4:**
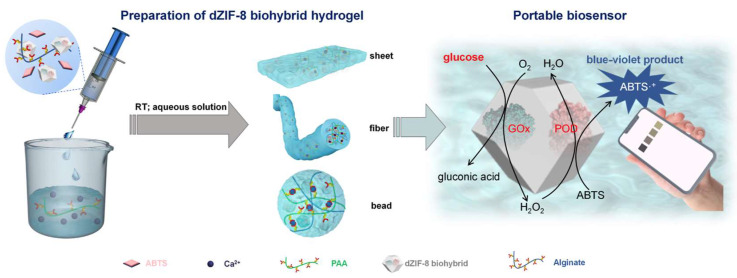
Schematic diagram of preparation of dZIG-8 biohybrid hydrogel and colorimetric sensing mechanism based on the biocatalytic cascade of dZIFs (reprinted with permission from [47]. Copyright 2022 American Chemical Society).

The encapsulation of sensing components, including nanomaterials or chromogenic reagents, is an effective strategy in the independent hydrogel platform. As shown in Figure 4, Zhong et al. developed a hybrid hydrogel-based biosensor encapsulating enzyme and defective metal−organic framework (MOF) for glucose detection [47]. Alginate was used as an encapsulation matrix and dual-crosslinked with the help of MOF. Because this hybrid hydrogel is stiff and highly stretchable, it could be fabricated in various forms, such as beads, fibers, or sheets. This study is important because it addresses the stability issue of both alginate and enzymes. The biohybrid hydrogel-based sensors detect glucose sensitively with an LoD of 0.05 mM. Also, they confirmed that 91.7% of sensing activity remained at room temperature for 30 days.

Particularly, hydrogels can provide a new opportunity for fluorescent probes. Lu et al. reported alginate hydrogel microsphere embedding CdZnTeS quantum dots (QDs) and urate oxide [48]. QD embedment into the hydrogel prevents the interference of background proteins, quenching of QD signals, and loss of enzyme activity. QDs are excellent and versatile optical nanomaterials, but they are not often limited to biomedical applications due to toxicity and immunogenicity. In addition, their inherent instability in biofluids with high-salt and protein-rich conditions often limits their potential. In this context, hydrogel encapsulation can prevent the direct exposure of QDs from samples. It is very important in terms of the shelf-life of the testing kit. Uric acid, a final product of purine metabolism requiring continuous and repetitive monitoring, can cause various diseases and syndromes when its level increases. In this study, the detection range of uric acid is between 100 μM and 900 μM and the LoD is estimated to be 20.3 μM. Similarly, hydrogels can be used as a matrix for upconversion nanoparticles (UCNPs), which convert near-infrared (NIR) irradiation into ultraviolet or visible emission. Su et al. fabricated a hydrogel suit for UCNPs to quantify carbaryl pesticide in the tea sample [49]. The UCNPs (NaErF_4_: 0.5% Tm^3+^@NaYF_4_), which showed monochromic red fluorescence, were encapsulated in the alginate hydrogel along with acetylthiocholine chloride (ATCh). Thereafter, hydrogels were exposed to the mixture consisting of acetylcholinesterase (AChE) and dopamine. Dopamine was polymerized and deposited on the UCNPs, thus quenching the fluorescence. In normal conditions without carbaryl, AChE turns ATCh to thiocholine (TCh) via a catalytic reaction, and TCh blocks the polymerization of dopamine on UCNPs, recovering red fluorescence. When the carbaryl was introduced into the system, the pesticide suppresses the activity of AChE to produce TCh. Thus, auto-polymerization of dopamine proceeded, and fluorescence was reduced according to the pesticide concentration. The LoD of this background-free system was 0.5 ng/mL and the detection range was in a range between 0.5 and 200 ng/mL.

In the meantime, Ning et al. reported a glow-type chemiluminescent (CL) sensor for cholesterol detection [50]. Glow-type CL, a subcategory of CL methods, has advantages over traditional flash-type CL sensors. Of note, longer CL time allows us to read results with the naked eye, which is an important characteristic in developing instrument-free analysis. The authors prepared the alginate hydrogels containing hydrogen peroxide, cholesterol oxide, and luminol. The role of the hydrogels here is to encapsulate the enzymes for enzymatic cascade reactions. This construction preserves enzymatic activities during long-term storage and also enables the generation of a longer and more stable luminescence. With smartphone-assisted detection, taking photographs in a homemade dark box, the LoD of the sensor was 7.2 μM, with a linear range between 0.01 and 0.35 mM.

Hydrogels can also be used as a final test strip. Yi et al. first developed and proved a scheme of Au@Ag nanostar-based detection of antioxidants and then later demonstrated inside the agarose-based test strip [51]. The core–shell Au@Ag nanostars were synthesized using the Ag etching process using HAuCl_4_, but this reaction is hampered by the presence of antioxidants. The differences in antioxidant concentration cause differences in particle morphology because of the degree of etching, varying the plasmonic properties of the resulting nanostars. The authors confirmed the usability of this mechanism via naked-eye detection and UV-vis spectroscopy measurement in the colloidal stage. Finally, they encapsulated this system in agarose-based test strips for practical use.

**Table 3:** Representative studies of polysaccharide hydrogel-based platforms for the development of biosensing platforms for point-of-care use.

Polysaccharide Hydrogel	Target Analyte	Sensing Components	Confirmation	Matrix	Detection Range	Limit of Detection	Response Time	Stability Test	Reference
Agarose	Glucose	Ag@Pt GOx, TMB	Spectrophotometer	Diluted Serum	5 to 55 μM	2.4 μM	N/A	12 h	2017 [52]
	Acetylcholine	Choline Oxidase, Mimic Peroxidase (Nanoflowers)	Smartphone	Tris-HCl Buffer	5 to 6000 μM	0.25 mM	60 m	15 d	2019 [53]
	Gallic acid	Au@Ag Core–Shell Nanostars	Smartphone	N/A	5.0 to 100 μM	9.82 μM	30 m	5 d	2023 [51]
	Cystine			N/A	5.0 to 100 μM	8.77 μM			
Alginate	Oxalate	MnO_2_/ TMB	Smartphone	Acetate Buffer	0.8 to 800 M	0.8 μM	10 m	7 d	2020 [54]
	Hepatitis B virus Surface Antigen (HBsAg)	ALP/ Pyrophosphate Ion/ anti-HBsAg Antibody	Smartphone	Buffer	1.56 to 50 mU/mL	0.24 ng/mL	30 m	N/A	2020 [55]
	Lactate	TiO_2_ Nanotube/LOx/GOx/HRP/TMB	Digital Camera	Artificial Sweat	0.1 to 1 mM	0.069 mM	4 m	10 d	2021 [56]
	Glucose				0.1 to 0.8 mM	0.044 mM	6 m		
	Cholesterol	HRP/COD/ Luminol	Smartphone	Isopropanol + Triton X-100	0.01 to 0.35 mM	7.2 μM	1 m	10 d	2021 [50]
	Uric Acid	CdZnTeS QDs/ Urate Oxidase	Smartphone	N/A	100 to 900 μM	20.3 μM	10 m	15 d	2021 [48]
			Spectrophotometer		1 to 100 μM	0.8 μM			
	Latate	Lactate Oxidase/HRP/TMB	Digital Camera	Artificial Sweat	10 to 100 mM	6.4 mM	13 m	N/A	2021 [57]
	Carbamate (Pesticide in Tea)	Upconversion NP/Dopamine/AChE/ATCh/	Smartphone	Tris-HCl Buffer	0.05 to 100 ng/mL	0.05 ng/mL	60 m	N/A	2021 [49]
	Paraoxon (Pesticide in Chinese Cabbages)	Au Nanoclusters -anchored MnO_2_/ AChE/ATCh/	Smartphone	Tris-HCl Buffer	5.0 to 500 ng/mL	5.0 ng/mL	65 m	10 d	2022 [58]
	Glucose	GOx/POD/PAA Defective ZIF-8	Smartphone	N/A	0.05 to 4 mM	0.05 mM	15 m	30 d	2022 [47]

Abbreviation: Glucose oxide (GOx), 3,3′,5,5′-tetramethylbenzidine (TMB), Horseradish peroxidase (HRP), 3,3′,5,5′-tetramethylbenzidine (TMB), Lactate oxidase (LOx), Quantum Dot (QD), Zeolitic imidazolate framework-8 (ZIF-8), Cholesterol oxidase (COD), Alkaline phosphatase (ALP), Poly(acrylic acid) (PAA), Peroxidase (POD), Not applicable (N/A).

## 4. Conclusions

In this review, we summarize recent studies of polysaccharide hydrogel-based biosensing platforms for POC use. Hydrogels, a certain class of biopolymers that can contain large amounts of liquid within a limited amount of solid components, have so far been applied to various biomedical and bioanalytical applications as support for enrichment or encapsulation matrices. In the POC testing format, such as PADs, microfluidic devices, or other independent platform, the integration of hydrogel provides various additional functions, such as hydrophilic coating, nanoscale filtration, stimuli-responsive behavior, signal enhancement, and biodegradation. Thus, this integration can help to address some critical issues of POC tests by improving sensitivity, selectivity, response time, or stability.

However, there are still many challenges ahead. Although polysaccharides, naturally occurring biopolymers, are abundant, biocompatible, and environmentally friendly, they have limitations in practical applications due to their inconsistency and instability. The first challenge for polysaccharide-based hydrogels is to achieve comparable levels to highly engineered synthetic hydrogels in terms of functionality and stability. Because the current status of the research remains at a laboratory scale, validation under practical conditions with large-scale analyses will be needed.

In the meantime, it is worth noting that more and more studies adopt smartphone technology for the sensing applications of the developed devices and platforms, as summarized in the tables above. In recent years, everyone has a smartphone, which is a portable telephone with an operating system, Internet access, computing power, developing/running application software, and a camera system. Hence, it can be utilized as a portable sensing instrument, having a user-friendly interface outside the laboratory. Furthermore, the role of smartphones in personal healthcare will become more important in the future with drastic technological developments (e.g., cloud computing and machine learning). We anticipate that its importance will increase as simpler formats are used in resource-limited settings. As the help of smartphones has brought about significant progress in PAD and microfluidics, it would be inseparable in the hydrogel-assisted biosensing platforms. Already, the introduction of smartphones enables us to operate two-way confirmation: a yes/no answer is simply confirmed by a naked eye readout, while quantitative analysis can be conducted with the help of a smartphone.

POC testing will be required everywhere in the future. The increased life expectancy emphasizes the importance of more frequent health monitoring on a personal and everyday basis. Under the threat of climate change, on-site detection of pollutants has become essential for environmental protection and a healthier life. In the meantime, the control of infectious diseases may also be a decisive factor in the future. As we witnessed during the COVID-19 pandemic, the crisis of infectious disease is not limited to a specific region; the catastrophic effects spread across the world immediately. Therefore, the development of new POC testing is highly entangled with the complex issues of our world, including industrialization, urbanization, and global equity. Therefore, the importance of affordable, rapid, rigid, equipment-free, and deliverable biosensing platforms made of cost-effective and environment-friendly materials will be highlighted more than ever. The water-absorbing properties, hydrophilicity, and versatile functionality of hydrogels are conceptionally interesting in the development of biosensing platforms for POC use. Going one step further, polysaccharide-based hydrogels that consist of common substances around us will provide an opportunity to realize this goal in a way that is economical and has less impact on the environment.

## Data Availability

Not applicable.

## References

[1] St-Louis P. (2000). Status of Point-of-Care Testing: Promise, Realities, and Possibilities. Clin. Biochem..

[2] Nichols J.H. (2007). Point of Care Testing. Clin. Lab. Med..

[3] Hayden O., Luppa P.B., Min J. (2022). Point-of-Care Testing—New Horizons for Cross-Sectional Technologies and Decentralized Application Strategies. Anal. Bioanal. Chem..

[4] Luppa P.B., Müller C., Schlichtiger A., Schlebusch H. (2011). Point-of-Care Testing (POCT): Current Techniques and Future Perspectives. TrAC Trends Anal. Chem..

[5] Gubala V., Harris L.F., Ricco A.J., Tan M.X., Williams D.E. (2012). Point of Care Diagnostics: Status and Future. Anal. Chem..

[6] Brendish N.J., Poole S., Naidu V.V., Mansbridge C.T., Norton N.J., Wheeler H., Presland L., Kidd S., Cortes N.J., Borca F. (2020). Clinical Impact of Molecular Point-of-Care Testing for Suspected COVID-19 in Hospital (COV-19POC): A Prospective, Interventional, Non-Randomised, Controlled Study. Lancet Respir. Med..

[7] Binnicker M.J. (2020). Challenges and Controversies to Testing for COVID-19. J. Clin. Microbiol..

[8] Kim Y.J., Min J. (2024). Advances in Nanobiosensors during the COVID-19 Pandemic and Future Perspectives for the Post-COVID Era. Nano Converg..

[9] Peppas N.A., Hilt J.Z., Khademhosseini A., Langer R. (2006). Hydrogels in Biology and Medicine: From Molecular Principles to Bionanotechnology. Adv. Mater..

[10] Calvert P. (2009). Hydrogels for Soft Machines. Adv. Mater..

[11] Robyt J.F. (1998). Essentials of Carbohydrate Chemistry.

[12] Chaudhary S., Jain V.P., Jaiswar G. (2022). Innovation in Nano-Polysaccharides for Eco-Sustainability.

[13] Ramesh H.P., Tharanathan R.N. (2003). Carbohydrates—The Renewable Raw Materials of High Biotechnological Value. Crit. Rev. Biotechnol..

[14] Yang X., Li A., Li X., Sun L., Guo Y. (2020). An Overview of Classifications, Properties of Food Polysaccharides and Their Links to Applications in Improving Food Textures. Trends Food Sci. Technol..

[15] Cazón P., Velazquez G., Ramírez J.A., Vázquez M. (2017). Polysaccharide-Based Films and Coatings for Food Packaging: A Review. Food Hydrocoll..

[16] Bu J., Lee T.H., Jeong W., Poellmann M.J., Mudd K., Eun H.S., Liu E.W., Hong S., Hyun S.H. (2020). Enhanced Detection of Cell-Free DNA (cfDNA) Enables Its Use as a Reliable Biomarker for Diagnosis and Prognosis of Gastric Cancer. PLoS ONE.

[17] Lee T., Rawding P.A., Bu J., Hyun S., Rou W., Jeon H., Kim S., Lee B., Kubiatowicz L.J., Kim D. (2022). Machine-Learning-Based Clinical Biomarker Using Cell-Free DNA for Hepatocellular Carcinoma (HCC). Cancers.

[18] Lee T.H., Jeon H.J., Choi J.H., Kim Y.J., Hwangbo P.-N., Park H.S., Son C.Y., Choi H.-G., Kim H.N., Chang J.W. (2023). A High-Sensitivity cfDNA Capture Enables to Detect the BRAF V600E Mutation in Papillary Thyroid Carcinoma. Korean J. Chem. Eng..

[19] Kim Y.J., Min J. (2023). Hydrogel-Based Technologies in Liquid Biopsy for the Detection of Circulating Clinical Markers: Challenges and Prospects. Anal. Bioanal. Chem..

[20] Kim Y.J., Cho Y.-H., Min J., Han S.-W. (2021). Circulating Tumor Marker Isolation with the Chemically Stable and Instantly Degradable (CSID) Hydrogel ImmunoSpheres. Anal. Chem..

[21] Negishi R., Takai K., Tanaka T., Matsunaga T., Yoshino T. (2018). High-Throughput Manipulation of Circulating Tumor Cells Using a Multiple Single-Cell Encapsulation System with a Digital Micromirror Device. Anal. Chem..

[22] Wei X., Tian T., Jia S., Zhu Z., Ma Y., Sun J., Lin Z., Yang C.J. (2016). Microfluidic Distance Readout Sweet Hydrogel Integrated Paper-Based Analytical Device (μDiSH-PAD) for Visual Quantitative Point-of-Care Testing. Anal. Chem..

[23] He G., Zhao S., Yang C., Chen L., Liu Y., Hu Q., Yang Y. (2024). Point-of-Care Monitoring of Milk Quality by Rapid Immunofluorescence with Mechanical Deformation of the Hydrogel Microspheres. Sens. Actuators B Chem..

[24] Wu Y., Li P., Yang L., Liu J. (2014). Individual SERS Substrate with Core–Satellite Structure Decorated in Shrinkable Hydrogel Template for Pesticide Detection. J. Raman Spectrosc..

[25] Qi G., Wang Y., Liu T., Sun D. (2023). “On-Site” Analysis of Pesticide Residues in Complex Sample Matrix by Plasmonic SERS Nanostructure Hybridized Hydrogel. Anal. Chim. Acta.

[26] Du G., Nie L., Gao G., Sun Y., Hou R., Zhang H., Chen T., Fu J. (2015). Tough and Biocompatible Hydrogels Based on in Situ Interpenetrating Networks of Dithiol-Connected Graphene Oxide and Poly(Vinyl Alcohol). ACS Appl. Mater. Interfaces.

[27] Kim Y.J., Min J. (2021). Property Modulation of the Alginate-Based Hydrogel via Semi-Interpenetrating Polymer Network (Semi-IPN) with Poly(Vinyl Alcohol). Int. J. Biol. Macromol..

[28] Nery E.W., Kubota L.T. (2013). Sensing Approaches on Paper-Based Devices: A Review. Anal. Bioanal. Chem..

[29] Hu J., Wang S., Wang L., Li F., Pingguan-Murphy B., Lu T.J., Xu F. (2014). Advances in Paper-Based Point-of-Care Diagnostics. Biosens. Bioelectron..

[30] Xu J., Khan H., Yang L. (2021). Hydrogel Paper-Based Analytical Devices: Separation-Free In Situ Assay of Small-Molecule Targets in Whole Blood. Anal. Chem..

[31] Wang W., Chen D., Cai Y., Liu Z., Yang H., Xie H., Liu J., Yang S. (2024). Sodium Alginate Hydrogelation Mediated Paper-Based POCT Sensor for Visual Distance Reading and Smartphone-Assisted Colorimetric Dual-Signal Determination of L -Lactate. Anal. Methods.

[32] Tang R.H., Li M., Liu L.N., Zhang S.F., Alam N., You M., Ni Y.H., Li Z.D. (2020). Chitosan-Modified Nitrocellulose Membrane for Paper-Based Point-of-Care Testing. Cellulose.

[33] Gabriel E.F.M., Garcia P.T., Cardoso T.M.G., Lopes F.M., Martins F.T., Coltro W.K.T. (2016). Highly Sensitive Colorimetric Detection of Glucose and Uric Acid in Biological Fluids Using Chitosan-Modified Paper Microfluidic Devices. Analyst.

[34] Choi J.R., Yong K.W., Tang R., Gong Y., Wen T., Yang H., Li A., Chia Y.C., Pingguan-Murphy B., Xu F. (2017). Lateral Flow Assay Based on Paper–Hydrogel Hybrid Material for Sensitive Point-of-Care Detection of Dengue Virus. Adv. Healthc. Mater..

[35] Lu F., Yang S., Ning Y., Wang F., Ji X., He Z. (2021). A Fluorescence Color Card for Point-of-Care Testing (POCT) and Its Application in Simultaneous Detection. Analyst.

[36] Xu J., Wang M., Li M., Yang J., Yang L. (2023). Paper-Based Chiral Biosensors Using Enzyme Encapsulation in Hydrogel Network for Point-of-Care Detection of Lactate Enantiomers. Anal. Chim. Acta.

[37] Linder V. (2007). Microfluidics at the Crossroad with Point-of-Care Diagnostics. Analyst.

[38] Erickson D., Li D. (2004). Integrated Microfluidic Devices. Anal. Chim. Acta.

[39] Hatch A., Hansmann G., Murthy S.K. (2011). Engineered Alginate Hydrogels for Effective Microfluidic Capture and Release of Endothelial Progenitor Cells from Whole Blood. Langmuir.

[40] Li Y., Yan X., Feng X., Wang J., Du W., Wang Y., Chen P., Xiong L., Liu B.-F. (2014). Agarose-Based Microfluidic Device for Point-of-Care Concentration and Detection of Pathogen. Anal. Chem..

[41] Lin Y.-H., Wang S.-H., Wu M.-H., Pan T.-M., Lai C.-S., Luo J.-D., Chiou C.-C. (2013). Integrating Solid-State Sensor and Microfluidic Devices for Glucose, Urea and Creatinine Detection Based on Enzyme-Carrying Alginate Microbeads. Biosens. Bioelectron..

[42] Goy C.B., Chaile R.E., Madrid R.E. (2019). Microfluidics and Hydrogel: A Powerful Combination. React. Funct. Polym..

[43] Liu X., Mei X., Yang J., Li Y. (2022). Hydrogel-Involved Colorimetric Platforms Based on Layered Double Oxide Nanozymes for Point-of-Care Detection of Liver-Related Biomarkers. ACS Appl. Mater. Interfaces.

[44] Yang S., Lu F., Liu Y., Ning Y., Tian S., Zuo P., Ji X., He Z. (2021). Quantum Dots-Based Hydrogel Microspheres for Visual Determination of Lactate and Simultaneous Detection Coupled with Microfluidic Device. Microchem. J..

[45] Garcia-Rey S., Gil-Hernandez E., Gunatilake U.B., Basabe-Desmonts L., Benito-Lopez F. (2023). Development of an Alginate/TiO2-Based Microfluidic Biosystem for Chrono-Sampling and Sensing of Glucose in Artificial Sweat. Sens. Actuators B Chem..

[46] Punjabi K., Adhikary R.R., Patnaik A., Bendale P., Saxena S., Banerjee R. (2022). Lectin-Functionalized Chitosan Nanoparticle-Based Biosensor for Point-of-Care Detection of Bacterial Infections. Bioconjug. Chem..

[47] Zhong N., Gao R., Shen Y., Kou X., Wu J., Huang S., Chen G., Ouyang G. (2022). Enzymes-Encapsulated Defective Metal–Organic Framework Hydrogel Coupling with a Smartphone for a Portable Glucose Biosensor. Anal. Chem..

[48] Lu F., Yang Y., Liu Y., Wang F., Ji X., He Z. (2021). Point-of-Care Testing (POCT) of Patients with a High Concentration of Uric Acid by Using Alginate Hydrogel Microspheres Embedded with CdZnTeS QDs and Urate Oxidase (Alg@QDs-UOx MSs). Analyst.

[49] Su D., Zhao X., Yan X., Han X., Zhu Z., Wang C., Jia X., Liu F., Sun P., Liu X. (2021). Background-Free Sensing Platform for on-Site Detection of Carbamate Pesticide through Upconversion Nanoparticles-Based Hydrogel Suit. Biosens. Bioelectron..

[50] Ning Y., Lu F., Liu Y., Yang S., Wang F., Ji X., He Z. (2021). Glow-Type Chemiluminescent Hydrogels for Point-of-Care Testing (POCT) of Cholesterol. Analyst.

[51] Yi J., Wang Z., Hu J., Yu T., Wang Y., Ge P., Xianyu Y. (2023). Point-of-Care Detection of Antioxidant in Agarose-Based Test Strip through Antietching of Au@Ag Nanostars. ACS Appl. Mater. Interfaces.

[52] Feng J., Huang P., Wu F.-Y. (2017). Gold–Platinum Bimetallic Nanoclusters with Enhanced Peroxidase-like Activity and Their Integrated Agarose Hydrogel-Based Sensing Platform for the Colorimetric Analysis of Glucose Levels in Serum. Analyst.

[53] Kong D., Jin R., Zhao X., Li H., Yan X., Liu F., Sun P., Gao Y., Liang X., Lin Y. (2019). Protein–Inorganic Hybrid Nanoflower-Rooted Agarose Hydrogel Platform for Point-of-Care Detection of Acetylcholine. ACS Appl. Mater. Interfaces.

[54] Jin R., Zhao L., Yan X., Han X., Liu M., Chen Y., Li Q., Su D., Liu F., Sun P. (2020). Lab in Hydrogel Portable Kit: On-Site Monitoring of Oxalate. Biosens. Bioelectron..

[55] Zheng W., Gao C., Shen L., Qu C., Zhang X., Yang L., Feng Q., Tang R. (2020). Alginate Hydrogel-Embedded Capillary Sensor for Quantitative Immunoassay with Naked Eye. Sensors.

[56] Gunatilake U.B., Garcia-Rey S., Ojeda E., Basabe-Desmonts L., Benito-Lopez F. (2021). TiO_2_ Nanotubes Alginate Hydrogel Scaffold for Rapid Sensing of Sweat Biomarkers: Lactate and Glucose. ACS Appl. Mater. Interfaces.

[57] Garcia-Rey S., Ojeda E., Gunatilake U.B., Basabe-Desmonts L., Benito-Lopez F. (2021). Alginate Bead Biosystem for the Determination of Lactate in Sweat Using Image Analysis. Biosensors.

[58] Li H., Zou R., Su C., Zhang N., Wang Q., Zhang Y., Zhang T., Sun C., Yan X. (2022). Ratiometric Fluorescent Hydrogel for Point-of-Care Monitoring of Organophosphorus Pesticide Degradation. J. Hazard. Mater..

